# Briarane Diterpenes from the South China Sea Gorgonian Coral, *Junceella gemmacea*

**DOI:** 10.3390/md12020589

**Published:** 2014-01-27

**Authors:** Wei Zhou, Jiao Li, Heng-Chao E, Bao-Shu Liu, Hua Tang, William H. Gerwick, Hui-Ming Hua, Wen Zhang

**Affiliations:** 1 Key Laboratory of Structure-Based Drug Design and Discovery, Ministry of Education; School of Traditional Chinese Materia Medica, Shenyang Pharmaceutical University, Shenyang 110016, China; E-Mail: zhouwei_0728@163.com; 2 Research Center for Marine Drugs, School of Pharmacy, Second Military Medical University, 325 Guo-He Road, Shanghai 200433, China; E-Mails: lijiao_2012@126.com (J.L.); ehengchao@126.com (H.-C.E.); liubaoshu@126.com (B.-S.L.); tanghua0309@126.com (H.T.); 3 Center for Marine Biotechnology and Biomedicine, Scripps Institution of Oceanography and Skaggs School of Pharmacy and Pharmaceutical Sciences, University of California San Diego, La Jolla, CA 92093, USA; E-Mail: wgerwick@ucsd.edu

**Keywords:** *Junceella gemmacea*, briarane, diterpenoid, junceellolide

## Abstract

Four new briarane diterpenoids, junceellolides M–P (**1**–**4**), were isolated together with seven known analogs (**5**–**11**) from the South China Sea gorgonian, *Junceella gemmacea*. The structures of these compounds were elucidated by detailed spectroscopic analysis and comparison with the reported data. The absolute configuration of compounds **1**–**3** were determined based on an ECD experiment, while the absolute configuration of compound **4** was genetically determined. All the compounds were isolated for the first time from *J. gemmacea*. These compounds showed no growth inhibitory activity against A549, MG63 and SMMC-7721 cell lines in an *in vitro* bioassay.

## 1. Introduction

Gorgonian corals of the genus *Junceella* (phylum, Cnidaria; class, Anthozoa; order, Gorgonacea; family, Ellisellidae) are widely distributed in the subtropical and tropical waters of the Indo-Pacific Ocean as whip-shaped unbranched colonies of variable colors. These animals are well-known as a source of highly oxidized diterpenes of the briarane class [1,2]. Briarane-related natural products are characterized by the presence of a γ-lactone fused to a bicyclo[8.4.0] ring system [3] and have attracted great attention due to their chemical diversity and wide spectrum of bioactivities, including cytotoxic [4], anti-inflammatory [5], antibacterial [6], immunomodulatory [7], anti-fouling [8] and insecticidal [9] effects. Chemical investigation on the genus of *Junceella* can be traced back to 1983, at which time the isolation of junceellin [10] was reported from the Chinese gorgonian *Junceella squamata* [1]. Since then, more than 140 briarane diterpenes have been isolated from members of this genus, which involves four species, namely *J*. *juncea*, *J*. *fragilis*, *J*. *gemmacea* and *J*. *squamata* [1,11]. Previous research activities have been focused on *J*. *juncea* and *J*. *fragilis*, and only a few reports have dealt with corals belonging to the species *J*. *gemmacea* and *J*. *squamata* [2].

**Figure 1:**
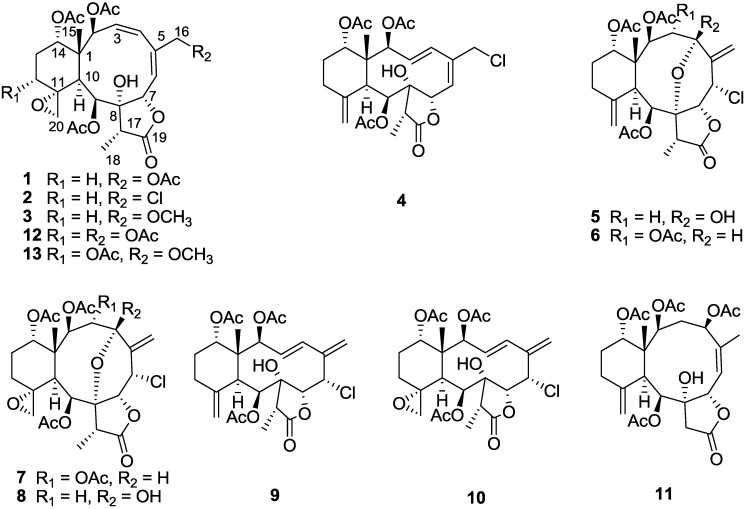
Structures of compounds **1**–**11**.

In our ongoing search for novel and bioactive secondary metabolites from marine invertebrates of the South China Sea, we were attracted by the potential medicinal value of the briarane diterpenoids. Previously, we reported the isolation of a series of briarane-type diterpenes with antimicrobial and tumor cell growth inhibition activities from the gorgonian, *Dichotella* *gemmacea* [12,13,14,15]. These results inspired us to continue to examine this class of metabolites, leading to the present investigation of the gorgonian *J. gemmacea* which was previously reported to be a rich source of briarane diterpenes. Chemical investigation on the acetone extract of these animals resulted in the isolation of four new briarane diterpenes, junceellolides M–P (**1**–**4**), together with seven known ones, namely junceellolide A (**5**), junceellin A (**6**), praelolide (**7**), juncin ZI (**8**), junceellolide B (**9**), junceellolide C (**10**) and junceellolide D (**11**) (Figure 1). The structures of these compounds were elucidated by extensive spectroscopic analysis (^1^H and ^13^C NMR, DEPT, HSQC, HMBC, NOESY, ^1^H–^1^H COSY and HRESIMS) and comparison with the reported analytical data for the known compounds. We herein report the isolation, structural determination and bioactivities of these new compounds.

## 2. Results and Discussion

Colonies of the gorgonian coral *J. gemmacea* (Valenciennes) were immediately frozen after collection and stored at −20 °C until extraction. The frozen organism was cut into small pieces and extracted exhaustively with acetone and methanol at room temperature. The Et_2_O-soluble portion (13.0 g) of the acetone extract was chromatographed repeatedly over silica gel, Sephadex LH-20, and semi-preparative RP-HPLC, to afford compounds **1**–**11**. By extensive spectroscopic analysis combined with careful comparison with the reported data, the structures of the known compounds were determined as junceellolide A (**5**) [16], junceellin A (**6**) [1,10], praelolide (**7**) [16], juncin ZI (**8**) [17], junceellolide B (**9**) [16], junceellolide C (**10**) [16] and junceellolide D (**11**) [16]. Junceellin A (**6**) and juncin ZI (**8**) were once reported from *J*. *squamata* and *J*. *juncea*, respectively, and their structures were elucidated on the basis of ESIMS, UV, IR and NMR techniques. The structures of junceellolides A–D (**5**, **9**–**11**) and praelolide (**7**), previously obtained from the South China Sea gorgonian, *J*. *fragilis*, were also established by extensive spectroscopic analysis.

Junceellolide M (**1**) was isolated as a white amorphous powder and had a molecular formula of C_28_H_36_O_12_ deduced from HRESIMS, indicating eleven degrees of unsaturation. The IR spectrum of **1** showed absorption bands for γ-lactone (1781 cm^−1^) and ester carbonyl (1736 cm^−1^) groups. This observation was consistent with the signals in the ^13^C NMR and DEPT spectra (Table 1) for nine sp^2^ carbon atoms (5 × OC=O, CH=CH, CH=C) at lower field and nineteen sp^3^ carbon atoms at higher field (1 × C, 2 × CH, 2 × CH_2_, 6 × CH_3_, 2 × OC, 4 × OCH, 2 × OCH_2_), accounting for seven double-bond equivalents. The remaining double bond equivalents were due to the presence of three rings in the molecule.

**Figure 2:**
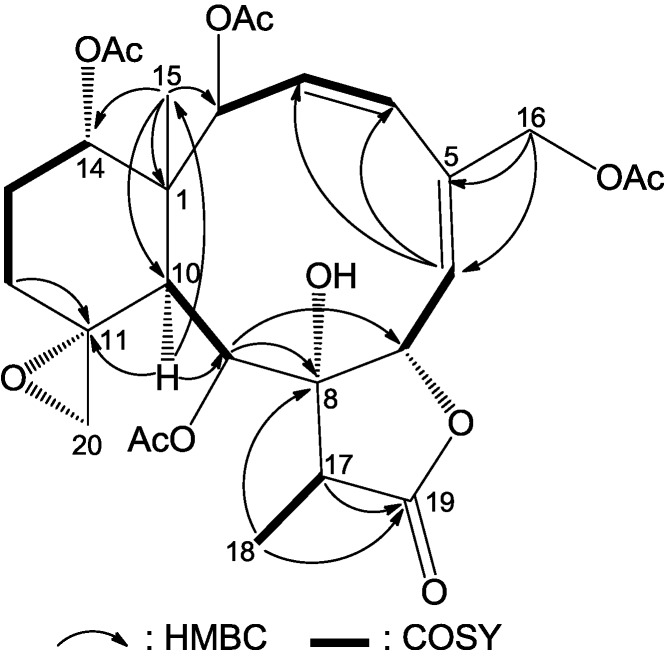
Key HMBC (arrow H→C) and ^1^H–^1^H COSY (bond) correlations for compound **1**.

The NMR data for compound **1** were almost identical to those of frajunolide D (**12**) [18], an analogue isolated from the gorgonian *J.* *fragilis*, except for the absence of one acetate ester group. Location of the missing acetate ester group to C-12 was indicated by the distinct correlations from H_2_-12 to H_2_-13 and to H-14 in a ^1^H–^1^H COSY experiment of **1** (Figure 2). The planar structure of **1** was further supported by ^1^H–^1^H COSY and HMBC data, as shown in Figure 2.

**Table 1:** ^1^H NMR data of compounds **1**–**4** (in CDCl_3_, *J* in Hz). s = singlet; d = doublet; t = triplet; m = multiplet; br s = broad singlet; br d = broad doublet; dd = doublet of doublets; tt = triplet of triplets; ov = overlapped signals.

Position	1 ^a^	2 ^b^	3 ^a^	4 ^a^
2	5.61 ov	5.52 d (9.5)	5.60 ov	5.61 ov
3	5.59 ov	5.61 dd (9.5, 10.5)	5.59 ov	6.16 dd (16.9, 5.7)
4	6.26 d (9.1)	6.33 d (10.5)	6.26 d (9.1)	6.41 d (16.9)
6	5.76 d (8.6)	6.01 d (9.0)	5.87 d (8.3)	5.62 ov
7	4.97 d (8.6)	4.95 dd (9.0, 1.5)	4.98 d (8.3)	5.69 br s
9	4.75 ov	4.75 d (5.5)	4.74 d (5.0)	5.94 d (5.7)
10	3.12 d (5.1)	3.10 d (5.5)	3.13 d (5.0)	3.28 d (5.7)
12α	2.20 ov	2.20 ov	2.20 ov	2.22 ov
12β	1.10 ov	1.10 ov	1.10 ov	2.25 ov
13β	1.73 m	1.73 m	1.72 ov	1.78 ov
13α	1.96 ov	1.94 ov	1.93 ov	1.95 ov
14	4.90 br s	4.89 br s	4.88 br s	4.88 br s
15-Me	1.00 s	1.01 s	1.00 s	0.90 s
16a	5.31 d (16.0)	4.62 d (13.0)	4.43 d (14.6)	4.16 ov
16b	4.72 d (16.0)	4.56 d (13.0)	4.27 d (14.6)	4.17 ov
17	2.27 q (7.0)	2.27 q (6.7)	2.26 q (7.0)	2.64 q (7.2)
18-Me	1.14 d (7.0)	1.13 d (6.7)	1.14 d (7.0)	1.25 d (7.2)
20a	3.50 br s	3.50 br s	3.51 br s	5.11 s
20b	2.65 d (2.5)	2.65 d (1.5)	2.64 d (2.2)	5.16 s
-OAc	2.18 s	2.18 s	2.18 s	2.25 s
	2.14 s	2.04 s	2.08 s	2.19 s
	2.09 s	1.99 s	1.98 s	2.10 s
	1.97 s			
-OH	2.85 d (1.7)	2.83 d (1.5)		
OMe			3.43 s (3H)	

^a^ Spectra recorded at 400 MHz; ^b^ spectra recorded at 500 MHz.

**Figure 3:**
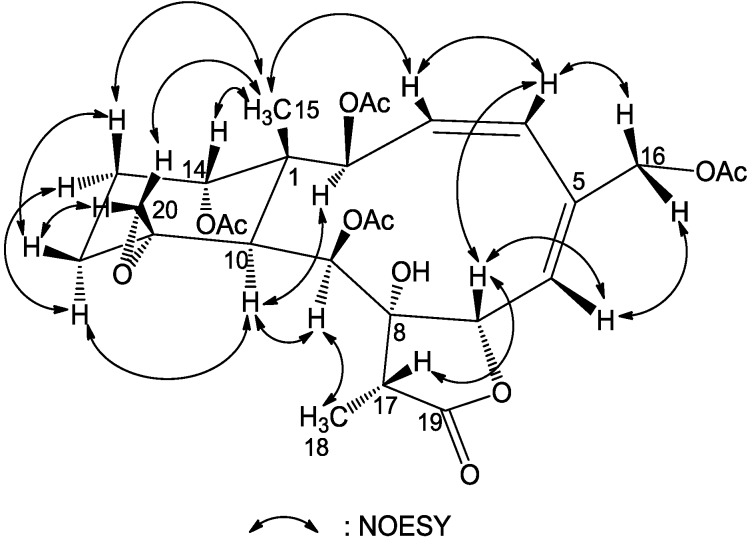
Key NOESY correlations for compound **1**.

The relative configuration of **1** was proven to be the same as that of frajunolide D (**12**) by a NOESY experiment (Figure 3), in which we have arbitrarily chosen the β*-*orientation of H-13β, H-14, Me-15, H-17 and H_2_-20 and an α-orientation of H-2, H-9, H-10, Me-18 and 8-OH. The geometry of the Δ^3^ double bond was assigned as *Z* based on the proton coupling constant between H-3 and H-4 (*J* = 8.7 Hz), while the geometry of the Δ^5^ double bond was *E* as deduced from an NOE correlation between H-6 and H-16a. The chemical shift value of C-11 and C-20 (δ_C_ 60.1 and 50.5, respectively) supported the α-configurational assignment of the 11,20-epoxy group. It has been reported that the ^13^C NMR shifts for C-11 and C-20 are at δ 62–63 and 58–60 when the epoxy group is β*-*oriented and at δ 55–61 and 47–52 ppm when α-oriented [19]. The relative configuration of **1** was thus determined as (1R*,2S*,3*Z*,5*E*,7S*,8S*,9S*,10S*,11R*,14S*,17R*).

**Figure 4:**
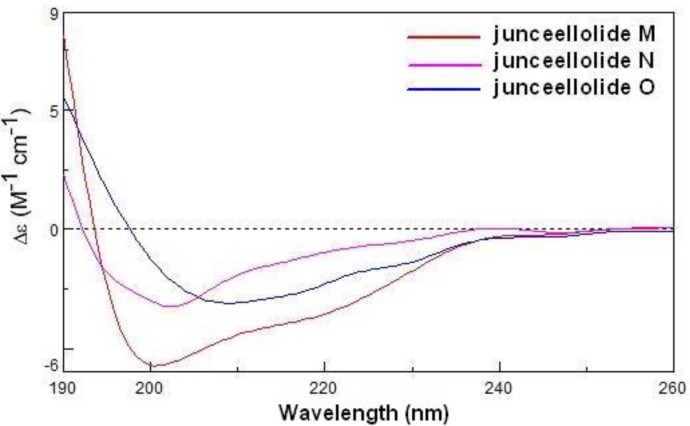
The ECD spectra of compounds **1**–**3** in acetonitrile.

Because compound **1** contained the same lactone and diene chromophores as gemmacolide N (**13**) [14], and because they differed only in the nature of the ester group at the C-12 and C-16 positions, the ECD spectrum of gemmacolide N could be used as an ECD reference for the configurational assignment of junceellolide M. Based on a negative ECD transition in the region 250–200 nm and a positive band below 200 nm, the absolute configuration of **1** was determined as (–)-(1*R*,2*S*,3*Z*,5*E*,7*S*,8*S*,9*S*,10*S*,11*R*,14*S*,17*R*) (Figure 4). 

Junceellolide N (**2**) was obtained as a white powder. The molecular formula was determined to be C_26_H_33_O_10_Cl by HRESIMS. An isotopic ratio of 3:1 was observed in the molecular ion peaks at *m/z* 563.1663/565.1782 [M + Na]^+^, further indicating the presence of one chlorine atom. The comparison of the overall ^1^H and ^13^C NMR data of **2** with those of **1** (Table 1) revealed a close similarity, except that the acetyl group at C-16 in **1** was replaced by a chlorine atom in **2**. The structure was fully supported by the 2D NMR experiments, including ^1^H–^1^H COSY, HMBC and NOESY. Compound **2** also gave a similar negative ECD band at 202 nm and a positive one below 200 nm (Figure 4), and thus its absolute configuration was then determined as (−)-(1*S*,2*S*,3*Z*,5*E*,7*S*,8*S*,9*S*,10*S*,11*R*,14*S*,17*R*).

Junceellolide O (**3**) had a molecular formula of C_27_H_36_O_11_, as deduced from the HRESIMS. The ^1^H and ^13^C NMR data of **3** also closely resembled those of **1**, except for the replacement of the acetyl group at C-16 in **1** by a methoxy group (δ_H_ 3.43, δ_C_ 58.5) in **3**. This assignment was further confirmed by the distinct HMBC correlation from H_2_-16 to the methoxy carbon (δ_C_ 58.5). The relative and absolute configuration of **3** was proven to be the same as that of **1** by analysis of the NOESY and ECD spectra, and was thus determined as (+)-(1*S*,2*S*,3*Z*,5*E*,7*S*,8*S*,9*S*,10*S*,11*R*,14*S*,17*R*). 

Junceellolide P (**4**) had a molecular formula of C_26_H_33_O_9_Cl, as determined by HRESIMS, and showed the presence of a chlorine atom in the molecule by the isotopic ratio of molecular ion peaks *m/z* 547.1715/549.1753 [M + Na]^+^ (3:1). Its IR spectrum indicated the presence of hydroxy (3497 cm^−1^), γ-lactone (1777 cm^−1^) and ester (1740 cm^−1^) functionalities. The ^1^H and ^13^C NMR spectra of **4** demonstrated a set of typical NMR signals for a briarane diterpenoid (Table 1), including ten sp^2^ carbon atoms (4 × OC=O, CH=CH, CH=C, CH_2_=C) at lower field and sixteen sp^3^ carbon atoms at higher field (1 × C, 2 × CH, 2 × CH_2_, 5 × CH_3_, 1 × OC, 4 × OCH, 1 × CH_2_Cl). 

**Figure 5:**
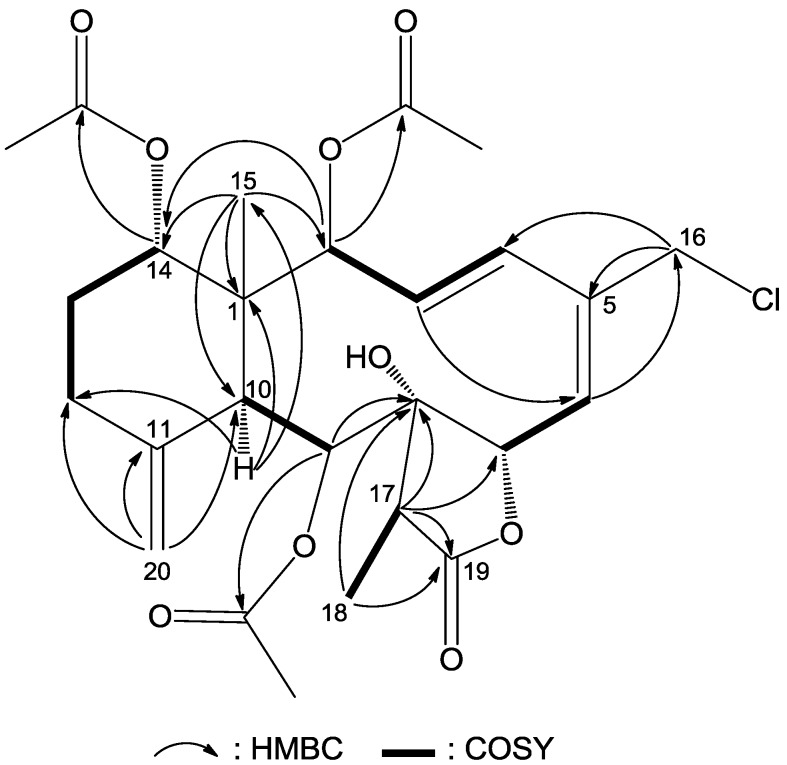
Key HMBC correlations (arrow H→C) and COSY (bond) spin coupling systems for compound **4**.

**Figure 6:**
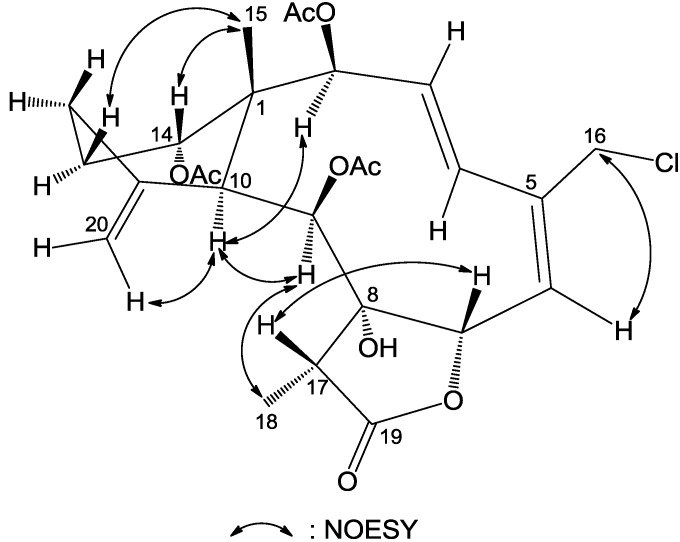
Key NOESY correlations of compound **4**.

The gross structure of compound **4** was characterized by a detailed analysis of 2D NMR spectra. The ^1^H–^1^H COSY spectrum gave five proton spin systems of H-2/H-3/H-4, H-6/H-7, H-9/H-10, H_2_-12/H_2_-13/H-14 and H-17/H_3_-18. The connections of these proton sequences led to the establishment of the planar structure of **4** by the observation of distinct HMBC correlations from H_3_-15 to C-1, C-2, C-10 and C-14, H_2_-16 to C-4 and C-5, H-6 to C-16, H-17 to C-7, C-8, C-9 and C-19, H-9 to C-8 and H_2_-20 to C-10, C-11 and C-12, respectively (Figure 5). The relative configurations at chiral centers were proven to be the same as those of compounds **1**–**3**. Interestingly, the large coupling constant for the olefinic protons (*J* = 16.8 Hz) indicated an *E* geometry of Δ^3^ in **4** in contrast to those of the *Z* double bonds in structures **1**–**3**. To our knowledge, this is the first report of a *trans* Δ^3,5^ conjugated diene in a briarane diterpenoid. The relative configuration of **4** was established by NOE (Figure 6) and coupling constant analysis whereas we propose its absolute configuration as (1*R*,2*S*,3*E*,5*E*,7*S*,8*S*,9*S*,10*S*,11*R*,14*S*,17*R*)-**4**, due to its biogenetic correlation with those of compounds **1**–**3**.

Compounds **1**–**11** were evaluated for their ability to inhibit tumor cell growth using the A549, MG63 and SMMC-7721 cell lines [20]. None of these compounds exhibited growth inhibitory effects against these cell lines in the *in vitro* bioscreening (IC_50_ ≥ 40 µM).

## 3. Experimental Section

### 3.1. General Experimental Procedures

Commercial silica gel (Yantai, China, 200–300; 400–500 mesh) and RP silica gel (Merck, Darmstadt, Germany, 43–60 µm) were used for column chromatography (CC). Sephadex LH-20 (GE Healthcare Bio-Sciences AB, SE-751 84 Uppsala, Sweden) was used for either purification or separation. TLC was carried out on precoated silica gel plates (Yantai, China, HSGF-254) and RP silica gel (Macherey-Nagel, Düren, Germany, RP-18 F254), and spots were detected on TLC under UV and visualized by spraying with anisaldehyde-sulfuric acid reagent, followed by heating. HPLC was performed using a system comprised of an Agilent G1311A pump, an Agilent G1315B DAD detector and a Rheodyne 7725 injection port. A semi-preparative normal-phase column (YMC Pack ODS-A, 250 × 10 mm I.D., particle size 5 µm, 250 × 10 mm, YMC Europe GmbH, Dinslaken, Germany) was used for HPLC. The NMR data were recorded on a Bruker DRX 400 and Avance 500 spectrometers at 400 or 500 MHz for ^1^H and 100 or 125 MHz for ^13^C, respectively. Chemical shifts are reported in parts per million (δ), with the use of the residual CDCl_3_ signal (δ_H_ = 7.27 ppm) as an internal standard for ^1^H NMR and CDCl_3_ (δ_C_ = 77.02 ppm) for ^13^C NMR, and the coupling constants (*J*) were in Hz. The ^1^H and ^13^C NMR assignments were supported by ^1^H–^1^H COSY, HSQC, HMBC and NOESY experiments. The following abbreviations are used to describe spin multiplicity: s denotes singlet; d denotes doublet; t denotes triplet; m denotes multiplet; br s denotes broad singlet; br d denotes broad doublet; dd denotes doublet of doublets; tt denotes triplet of triplets; ov denotes overlapped signals. Optical rotations were measured in CH_2_Cl_2_ with an Autopol IV polarimeter at the sodium D line (590 nm). Infrared spectra were recorded in thin polymer films on a Nexus 470 FT-IR spectrophotometer (Nicolet, USA); peaks are reported in cm^−1^. UV absorption spectra were recorded on a Varian Cary 100 UV-Vis spectrophotometer; peak wavelengths are reported in nanometers. Circular dichroism spectra were recorded with a JASCO J-715 circular dichroism spectropolarimeter. The HRMS were performed on a Q-TOF micro-mass spectrometer (resolution: 5000). An isopropyl alcohol solution of sodium iodide (2 mg/mL) was used as a reference compound. 

### 3.2. Animal Material

Specimens of the gorgonian coral *Junceella gemmacea* were collected from the South China Sea in October 2011, and identified by Xiu-Bao Li of the South China Sea Institute of Oceanology, Academia Sinica. The voucher specimen was deposited in the Second Military Medical University, Shanghai, China.

### 3.3. Extraction and Isolation

The frozn animals (2500 g, wet weight) were cut into small pieces and extracted ultrasonically for four times (3000 mL × 4) with acetone and MeOH at room temperature. The organic extracts were concentrated under vacuum to give a residue, which was suspended into H_2_O and extracted with diethyl ether and *n*-butanol for four times, respectively. The Et_2_O-soluble portion was separated on a Sephadex LH-20 column (CH_2_Cl_2_:MeOH 1:1) giving fractions 1–10 (Fr.1~Fr.10). Fr.6 was subjected to SiO_2_ column chromatography using solvents of increasing polarity from petroleum to acetone (TLC (GF 254) monitoring) to obtain eleven subfractions (P.1~P.11). P.11 was separated by reversed-phase silica gel chromatography (gradient MeOH/H_2_O, from 30:70 to 0:100), followed by semi-preparative RP-HPLC (MeOH/H_2_O, 58:42, flow rate of 2.0 mL/min), to yield compounds **1** (4.2 mg, *t*_R_ 35 min) and **3** (3.7 mg, *t*_R_ 32 min). Fr.8 was fractioned on silica gel eluting with petroleum/acetone (stepwise, 20:1–0:1) and then chromatographed on a gravity column with ODS using MeOH/water (gradient from 30:70 to 0:100, in 10% increments) as the eluent to afford subfractions S.10 and S.11. S.10 was isolated by reversed-phase silica gel chromatography (gradient elution from MeOH/H_2_O (30:70) to 100% MeOH, in 10% increments) and successively subjected to RP-HPLC (MeOH/H_2_O 79:21, 2 mL/min), producing **5** (2.6 mg, *t*_R_ 29 min). S.11 was chromatographed over RP-silica gel column chromatography using a gradient of MeOH/H_2_O (30:70 to 0:100, in 10% increments) to yield subfractions R.4 and R.5. A HPLC chromatographic isolation on R.4 (MeOH/H_2_O 62:38, 2 mL/min) led to the preparation of **2** (2.4 mg, *t*_R_ 60 min), **8** (2.3 mg, *t*_R_ 39 min), **10** (4.9 mg, *t*_R_ 53 min) and **11** (3.4 mg, *t*_R_ 83 min). Furthermore, R.5 was further separated by HPLC using MeOH/H_2_O (56:44) at the flow rate of 2.0 mL/min and provided **4** (5.9 mg, *t*_R_ 51 min) and **9** (1.6 mg, *t*_R_ 54 min). Using petroleum/acetone (7:1) as the eluent, the crystal precipitated from Fr.8 furnished **6** (35.0 mg) and **7** (16.9 mg) on Silica gel column chromatography.

**Junceellolide M (1)**: white amorphous powder; 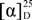 = −0.85° (*c* 0.47, CH_2_Cl_2_); UV (MeOH) 230 nm; CD (CH_3_CN, *c* 3.0 × 10^−4^) λ_max_ (Δε) positive below 193.5 nm, 200 (−5.67) nm; IR (film) ν_max_ 2959, 2930, 1781, 1736, 1252, 1225 cm^−1^; ^1^H NMR spectroscopic data, see Table 1; ^13^C NMR spectroscopic data, see Table 2; HRESIMS *m/z* 587.2100 [M + Na]^+^, calcd. for C_28_H_36_O_12_Na, 587.2104. 

**Table 2:** ^13^C NMR data of compounds **1**–**4** (in CDCl_3_, *J* in Hz).

Position	1 ^a^	2 ^b^	3 ^a^	4 ^a^
1	47.1, C	47.2, C	47.3, C	49.1, C
2	74.7, CH	74.6, CH	74.8, CH	75.5, CH
3	132.6, CH	132.3, CH	131.7, CH	137.8, CH
4	127.2, CH	127.8, CH	128.2, CH	126.4, CH
5	139.6, C	140.0, C	141.4, C	140.0, C
6	122.5, CH	126.2, CH	123.0, CH	127.6, CH
7	78.8, CH	78.6, CH	79.0, CH	80.7, CH
8	80.9, C	80.9, C	80.9, C	80.7, C
9	64.6, CH	64.6, CH	64.7, CH	73.4, CH
10	37.8, CH	37.8, CH	37.8, CH	43.2, CH
11	60.1, C	60.0, C	60.1, C	148.9, C
12	29.2, CH_2_	29.2, CH_2_	29.3, CH_2_	31.2, CH_2_
13	25.1, CH_2_	25.0, CH_2_	25.1, CH_2_	27.8, CH_2_
14	74.4, CH	74.5, CH	74.7, CH	78.8, CH
15	14.4, CH_3_	14.4, CH_3_	14.5, CH_3_	15.7, CH_3_
16	63.0, CH_2_	44.9, CH_2_	72.4, CH_2_	47.5, CH_2_
17	44.0, CH	44.0, CH	44.1, CH	45.4, CH
18	6.4, CH_3_	6.4, CH_3_	6.4, CH_3_	8.2, CH_3_
19	175.6, C	175.6, C	175.7, C	175.4, C
20	50.5, CH_2_	50.5, CH_2_	50.5, CH_2_	111.4, CH_2,_
-OAc	170.3, C	170.2, C	170.3, C	170.4, C
	21.6, CH_3_	21.6, CH_3_	21.6, CH_3_	21.5, CH_3_
	170.2, C	170.2, C	170.2, C	169.7, C
	20.9, CH_3_	21.3, CH_3_	21.3, CH_3_	21.7, CH_3_
	170.4, C	169.7, C	169.5, C	169.9, C
	21.2, CH_3_	21.1, CH_3_	21.2, CH_3_	20.9, CH_3_
	169.4, C			
	21.1, CH_3_			
OMe			58.5, CH_3_	

^a^ Spectra recorded at 100 MHz; ^b^ spectra recorded at 125 MHz.

**Junceellolide N (2)**: white amorphous powder; 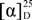 = −10.89° (*c* 0.32, CH_2_Cl_2_); UV (MeOH) 418, 231 nm; CD (CH_3_CN, *c* 3.0 × 10^−4^) λ_max_ (Δε) positive below 192 nm, 202.5 (−3.23) nm; IR (film) ν_max_ 2959, 2930, 1782, 1731, 1288, 1274 cm^−1^; ^1^H NMR spectroscopic data, see Table 1; ^13^C NMR spectroscopic data, see Table 2; HRESIMS *m/z* 563.1663 [M + Na]^+^, calcd. for C_26_H_33_O_10_ClNa, 563.1660.

**Junceellolide O (3)**: white amorphous powder; 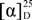 = +8.75° (*c* 0.04, CH_2_Cl_2_); UV (MeOH) 228 nm; CD (CH_3_CN, *c* 3.0 × 10^−4^) λ_max_ (Δε) positive below 198 nm, 209 (−3.08) nm; IR (film) ν_max_ 2959, 2930, 1780, 1732, 1272 cm^−1^; ^1^H NMR spectroscopic data, see Table 1; ^13^C NMR spectroscopic data, see Table 2; HRESIMS *m/z* 559.2158 [M + Na]^+^, calcd. for C_27_H_36_O_11_Na, 559.2155.

**Junceellolide P (4)**: white acicular crystal, 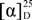 = −59.29° (*c* 0.28, CH_2_Cl_2_); UV (MeOH) 229 nm; IR (film) ν_max_ 3456, 1777, 1740, 1236 cm^−1^; ^1^H NMR spectroscopic data, see Table 1; ^13^C NMR spectroscopic data, see Table 2; HRESIMS *m/z* 547.1715 [M + Na]^+^, calcd. for C_26_H_33_O_9_ClNa, 547.1711.

### 3.4. Cytotoxicity Assay

Compounds **1**–**11** were evaluated for cytotoxicity against human lung adenocarcinoma (A549), human osteosarcoma cell (MG63) and human hepatocellular carcinoma cell lines (SMMC-7721), using a modification of the 3-(4,5-dimethylthiazol-2-yl)-2,5-diphenyltetrazolium bromide (MTT) colorimetric method [21]. Adriamycin was used as a positive control.

## 4. Conclusions

Chemical investigation of the South China Sea gorgonian, *Junceella gemmacea* (Valenciennes), has led to the isolation of four new 3,5-dien-7,18-olide briarane-type diterpenoids, junceellolides M–P (**1**–**4**), along with seven previously described analogues (**5**–**11**). All the compounds were reported for the first time from *Junceella gemmacea*. The compounds were tested for growth inhibition activity against A549, MG63 and SMMC-7721 cell lines but were found to be inactive.

The isolation of an array of briarane diterpenoids demonstrates the productive chemical diversity of the gorgonian coral *J. gemmacea*, similar to that found in other species of this genus as well as in the genus *Dichotella* [2,20,22] The complexity of these intriguing structures and variable potential for tumor cell growth inhibition activity of compounds in this structure class may attract additional attention from chemists and pharmacologists, and encourage further investigations on the chemistry and antitumor activity of this cluster of metabolites.
